# Dietary 1,3-β-Glucans Affect Growth, Breast Muscle Composition, Antioxidant Activity, Inflammatory Response, and Economic Efficiency in Broiler Chickens

**DOI:** 10.3390/life13030751

**Published:** 2023-03-10

**Authors:** Shimaa A. Amer, Amany Behairy, Ahmed Gouda, Abdel-Wahab A. Abdel-Warith, Elsayed M. Younis, Elshimaa M. Roushdy, Amr A. Moustafa, Noura A. Abd-Allah, Rehab Reda, Simon J. Davies, Seham M. Ibrahim

**Affiliations:** 1 Department of Nutrition and Clinical Nutrition, Faculty of Veterinary Medicine, Zagazig University, Zagazig 44511, Egypt; sehamyousef2011@yahoo.com; 2 Physiology Department, Faculty of Veterinary Medicine, Zagazig University, Zagazig 44511, Egypt; amanyshoaib43@gmail.com; 3 Animal Production Department, Agricultural & Biological Research Division, National Research Center, Dokki, Cairo 11865, Egypt; black_tiger2167@yahoo.com; 4 Department of Zoology, College of Science, King Saud University, P.O. Box 2455, Riyadh 11451, Saudi Arabia; awarith@ksu.edu.sa (A.-W.A.A.-W.); emyounis@ksu.edu.sa (E.M.Y.); 5 Animal Wealth Development Department, Faculty of Veterinary Medicine, Zagazig University, Zagazig 44511, Egypt; shimaa_production@yahoo.com (E.M.R.); rehabreda268@yahoo.com (R.R.); 6 Department of Biochemistry, Faculty of Veterinary Medicine, Zagazig University, Zagazig 44511, Egypt; amr.moustafa@gmx.de; 7 Clinical Pathology Department, Faculty of Veterinary Medicine, Zagazig University, Alzeraa Street, Zagazig 44511, Egypt; omarfahd334@gmail.com; 8 School of Science and Engineering, National University of Ireland Galway Republic of Ireland, H91 TK33 Galway, Ireland; sjdplymouth@live.co.uk

**Keywords:** broilers, 1,3-β-glucans, growth, bird’s health, immunohistochemistry

## Abstract

Recently, researchers have been intensively looking for novel, safe antibiotic alternatives because of the prevalence of many clinical and subclinical diseases affecting bird flocks and the risks of using antibiotics in subtherapeutic doses as feed additives. The present study intended to evaluate the potential use of 1,3-β-glucans (GLC) as antibiotic alternative growth promotors and assessed the effect of their dietary inclusion on the growth performance, carcass traits, chemical composition of breast muscles, economic efficiency, blood biochemical parameters, liver histopathology, antioxidant activity, and the proinflammatory response of broiler chickens. This study used 200 three-day-old ROSS broiler chickens (50 chicks/group, 10 chicks/replicate, with an average body weight of 98.71 ± 0.17 g/chick). They were assigned to four experimental groups with four dietary levels of GLC, namely 0, 50, 100, and 150 mg kg^−1^, for a 35-day feeding period. Birds fed diets containing GLC showed an identical different growth rate to the control group. However, the total feed intake (TFI) increased quadratically in the GLC50 and GLC100 groups as compared to that in the control group. GLC addition had no significant effect on the weights of internal and immune organs, except for a decrease in bursal weight in the GLC150 group (*p* = 0.01). Dietary GLC addition increased the feed cost and total cost at 50 and 100 mg kg^−1^ doses. The percentages of n-3 and n-6 PUFA in the breast muscle of broiler chickens fed GLC-supplemented diets increased linearly in a dose-dependent manner (*p* < 0.01). The serum alanine aminotransferase (ALT) level and the uric acid level were quadratically increased in the GLC150 group. The serum levels of total antioxidant capacity, catalase, superoxide dismutase, interleukin-1β, and interferon-gamma linearly increased, while the MDA level decreased in the GLC-fed groups in a dose-dependent manner. Normal histological characterization of different liver structures in the different groups with moderate round cells was noted as a natural immune response around the hepatic portal area. The different experimental groups showed an average percentage of positive immunostaining to the proinflammatory marker transforming growth factor-beta with an increase in the dose of GLC addition. The results suggest that GLC up to 100 mg kg^−1^ concentration can be used as a feed additive in the diets of broiler chickens and shows no adverse effects on their growth, dressing percentage, and internal organs. GLC addition in diets improves the antioxidant activity and immune response in birds. GLC help enrich the breast muscle with n-3 and n-6 polyunsaturated fatty acids.

## 1. Introduction

The economic production of broiler chickens is critical for sustained meat supply, and feed is a crucial factor that influences the economic and productive performance of broiler chickens [1,2]. Antibiotic supplements are used in animal diets to boost their growth, feed efficiency, and disease resistance. Irrational use of antibiotics in animal feed has resulted in an increase in antibiotic-resistant bacteria, thereby endangering human health and influencing the natural growth of livestock production. Since the prohibition of antibiotic use as a feed additive in 2006 by the European Union, there is an urgent need to investigate and broaden the range of antibiotic alternatives [3]. Several potential immune modulators may act as substitutes for antibiotics used in animal production to promote growth and disease resistance [4,5,6]. Specific feed ingredients in poultry diets improve digestion by creating a favorable gut environment [2]. Different feed additives can now be used as growth promoters instead of antibiotics [4,7].

β-glucans (GLC) are glucan polymers derived from the cell walls of yeast, fungi, and bacteria as well as cereal grains such as barley and oat [8]. 1,3 β-glucans are chains of glucose molecules linked together by β-glycosidic bonds between carbons #1 and #3. Significant structural variations in GLC from these sources lead to differences in their physiological mechanisms [9]. Because of their highly branched structure, GLC derived from yeast and fungal sources are the most efficient for boosting immunity against pathogenic organisms [10,11]. These GLC can reduce coccidia infection in growing pullets and laying hens [12]. GLC can boost both innate and adaptive immune responses [8].

The addition of GLC to feed enhanced gut health and disease resistance in birds challenged with necrotic enteritis by stimulating the gene expression of antimicrobial peptides [13]. By enhancing the populations of cells secreting IgA and goblet cells, yeast-based GLC can serve as growth inducers and prospective antibiotic alternatives against specific microorganisms in birds [14]. Moreover, macrophages obtained from chickens fed a diet supplemented with GLC showed upregulated expression of IL-1 [15], IL-2, interferon (IFN)-γ [16], IL-4, and IL-18 [17]. Additionally, yeast GLC boosted humoral and cell-mediated immune responses in broilers [8,18]. Other studies have found that yeast GLC are absorbed through the intestinal mucosa and promote the innate immune system, thereby causing a higher production of macrophages, monocytes, and cytokines and a lower susceptibility to diseases [8,19]. GLC supplementation increased lysozyme and complement levels, increased CD3 and CD20 expression, altered tight junction protein expression, and improved phagocytic activity [20,21]. 

In the present study, we used algae-based GLC produced from a unique species of microalgae (*Euglena gracilis*) with more than 50% content of 1,3 β-glucan, unlike yeast-based GLC that contain 5–15% 1,3 β-glucan. The high digestibility of algal cells makes GLC more bioavailable without extraction. Therefore, the aim of the present study was to assess the potential effects of the dietary addition of algae-based GLC as antibiotic alternative growth promoters on some production performance and health aspects of broiler chickens such as growth, carcass traits, economic efficiency, chemical composition of breast muscles, blood biochemical parameters, liver histopathology, antioxidant activity, and proinflammatory responses in broiler chickens.

## 2. Materials and Methods

### 2.1. Birds, Experimental Design, and Diets

The experimental protocol received ethical approval from the Institutional Animal Care and Use Committee of Zagazig University in Egypt (approval no. ZU-IACUC/2/F/153/2022).

A total of 200 one-day-old mixed chicks (Ross 308 broiler) were obtained from a commercial chick producer (Al-Abrar Hatchery, Abou Hammad, Al Sharqia Governorate, Egypt). Chicks were reared in an open, well-ventilated house with sawdust bedding (7 birds/m^2^). The experiment lasted for 35 days. During the first week, the building temperature was set at 34 °C and gradually reduced until it reached 25 °C at the end of the experiment. The illumination regime was set to 23:1 h light/dark, then 20:4 h light/dark until the end of the experiment. The birds were exposed to a 3-day adaptation period prior to the experiment to achieve an average body weight (BW) of 98.71 ± 0.17 g. They were then randomly assigned to four treatment groups with five replicates for each group (10 chicks/replicate). Chicks were fed basal diets supplemented with four concentrations of GLC (Aleta^TM^, Kemin Industries, Scott Ave Des Moines, IA, USA): 0, 50, 100, and 150 mg kg^−1^ of diet. The treatment groups were designated as GLC0, GLC50, GLC100, and GLC150, respectively. The birds were fed the experimental diets on three feeding periods: starter (4th–10th day), grower (11th–23rd day), and finisher period (24th–35th day). The GLC was mechanically mixed with other feed ingredients and offered to the birds in a mash form. The proximate composition of the basal diet is shown in Table 1. The experimental diets and bird management conditions were designed in accordance with the Ross 308 broiler nutrition specifications, AVIAGEN [22]. The birds that remained after the study were released.

### 2.2. Growth Performance

Chickens were singly weighed on the 4th day of life to determine the average initial BW. BW, BW gain (BWG), and feed intake (FI) were then recorded at 10, 23, and 35 days. The feed conversion ratio (FCR) was calculated by dividing FI by BWG.

The protein efficiency ratio (PER) was calculated according to McDonald et al. [23].

(1) $$\mathrm{PER}=\frac{\mathrm{Live}\;\mathrm{weight}\;\mathrm{gain}\;\left(g\right)\;}{\mathrm{Protein}\;\mathrm{intake}\;\left(g\right)}$$

The relative growth rate (RGR) was calculated according to Brody [24].

(2) $$\mathrm{RGR}=\frac{W2-W1}{0.5\left(W1+W2\right)}\times 100$$

where W1 = initial body weight (g) and W2 = final body weight (g). 

### 2.3. Sampling

At the end of the feeding period, the birds (10 birds/group) were euthanized by cervical dislocation [25]. The blood was collected in plain centrifuge tubes without an anticoagulant and centrifuged at 3000 rpm for 15 min to separate serum [26]. Samples from the breast muscle were collected for fatty acid analysis and proximate composition determination. The serum samples were stored in a deep freezer at −20 °C until physicochemical analysis. We collected liver samples for histological analysis and liver and spleen samples for immunohistochemistry.

### 2.4. Carcass, Gut, and Immune Organ Characteristics

Ten birds from each group were randomly chosen, fasted, and euthanized by cervical dislocation [25]. The weight of hot carcass was determined. The plucked and eviscerated carcasses were stripped of their feet; feathers; heads; and internal organs such as the liver, heart, gizzard, bursa of Fabricus, spleen, and digestive system. The carcass weight was determined, and the dressing percentage was estimated as follows:

(3) $$\mathrm{Dressing}\%=\frac{\mathrm{Carcass}\;\mathrm{weight},\;g}{\mathrm{Live}\;\mathrm{BW},\;g}\times 100$$

### 2.5. Fatty Acid Profile and Chemical Composition of Breast Muscles

At the end of the feeding period, five breast muscle samples were collected from each group. The proximate composition of the breast muscles (percentage content of dry matter, crude protein, fat, and ash) was determined. We also extracted the oils from the breast muscle by using a solvent mixture of chloroform/methanol (2:1, *v*/*v*) for fatty acid analysis [27]. The chemical composition of the breast muscles and fatty acid profile in the extracted oil were determined following the method of AOAC [28].

### 2.6. Blood Biochemical Indices

An automatic biochemical analyzer (Robonik Prietest ECO, Navi Mumbai, India) was used to measure serum creatinine and uric acid levels [29,30]. We also measured the serum levels of alanine aminotransferase (ALT) and aspartate aminotransferase (AST) [31].

### 2.7. Antioxidant Activity and Inflammatory Indices

The serum malondialdehyde (MDA) level was measured according to the method of Mcdonald and Hultin [32]. We also measured serum superoxide dismutase (SOD) activity [33], catalase (CAT) [34], and total antioxidant capacity (TAC) [35]. Specific ELISA assay kits (MyBioSource, San Diego, CA, USA; Cat. No. MBS700243 and MBS2024496) were used to measure interferon-gamma (IFN-γ) and interleukin-1β (IL-1β) levels, respectively. 

### 2.8. Liver Histology

Following the method of Omar et al. [5], samples were extracted from the chicken’s liver and fixed in neutral buffered formalin (10%) for the histopathological study. 

### 2.9. Immunohistochemistry

The inflammatory response of broiler chickens fed GLC was investigated in the leucocytic populations of the liver and spleen by determining TGF-β levels according to the methods described by Amer et al. [36].

### 2.10. Economic Efficiency

The feed cost (FC), total cost (TC), total return (TR), and net profit (NP) were calculated according to the method of El-Telbany and Atallah [37] and Dunning and Daniels [38] as follows:

TC (USD) = Fixed cost + Variable cost (FC, USD);

FC/kg gain = Total FC/Total BWG;

TR (USD)/bird = Price of kg × live BW/bird;

NP (USD) = TR − TC;

Economic efficiency (EE) = NP (USD)/total FC (USD).

Performance index% (PI) was calculated according to the method of North and Bell [39] as follows:

PI% = final live BW (kg)/FCR × 100.

### 2.11. Statistical Analysis

The normality of the data was verified using the Shapiro–Wilk test, and the homogeneity of variance among the components of the experimental treatments was confirmed by Levene’s test. The data were analyzed using one-way analysis of variance (ANOVA) and the GLM procedure in SPSS (SPSS Inc., Chicago, IL, USA). By using orthogonal polynomial contrasts, the linear and quadratic effects of GLC were demonstrated. Tukey’s test was used to compare mean differences with a 5% probability. The data variation was represented as pooled SEM, and the significance level was established at *p* < 0.05. 

## 3. Results

### 3.1. Growth Performance

Table 2 shows the growth parameters of broiler chickens fed on the experimental diets. During the starter, growth, and finisher periods, there was no significant effect of the dietary addition of GLC with different levels on BW, BWG, and FCR of chickens (*p* > 0.05). The only exception was the feed intake that quadratically increased in the GLC100 group during the grower period and in the GLC50 group during the finisher period relative to the GLC0 group (*p* < 0.01). The overall performance was nonsignificant among the groups for final BW, total BWG, FCR, RGR, and PER (*p* > 0.05). The total feed intake was quadratically augmented in the GLC50 and GLC100 groups as compared to that in the control group (GLC0) (*p* < 0.01).

### 3.2. Carcass, Gut, and Immune Organ Characteristic 

GLC supplementation showed no significant effects on the dressing percentage and intestine, spleen, gizzard, and liver weight (*p* > 0.05). However, the bursal weight was decreased in the GLC150 group (*p* = 0.01) (Table 3).

### 3.3. Composition and Fatty Acid Profile of Breast Muscles

Dietary GLC supplementation did not significantly affect the dry matter, crude protein, fat, and ash content of breast muscles (*p* > 0.05). The breast muscles of birds fed GLC-supplemented diets showed a linear increase in the percentages of α-linolenic acid, eicosapentaenoic acid, docosapentaenoic acid, docosahexaenoic acid, linoleic acid, arachidonic acid, n-3 PUFA, and n-6 PUFA (*p* < 0.01) (Table 4).

### 3.4. Serum Biochemical Parameters

The liver and kidney function test results represented by the serum levels of ALT, AST, uric acid, and creatinine are shown in Table 5. The ALT level was quadratically increased in the GLC150 group (*p* = 0.03). The GLC150 group showed a linear increase in uric acid levels (*p* = 0.03). The serum AST and creatinine levels did not differ significantly between the groups. 

### 3.5. Antioxidant Capacity and Inflammatory Indices

The effects of GLC supplementation on the antioxidant and inflammatory indices are shown in Table 6. The serum TAC, CAT, SOD, IL-1β, and IFN-γ levels linearly increased in the GLC-fed groups (*p* < 0.01). In contrast, the MDA levels were linearly reduced in the GLC-fed groups (*p* < 0.01). 

### 3.6. Histological Examination of the Liver

**Figure 1:**
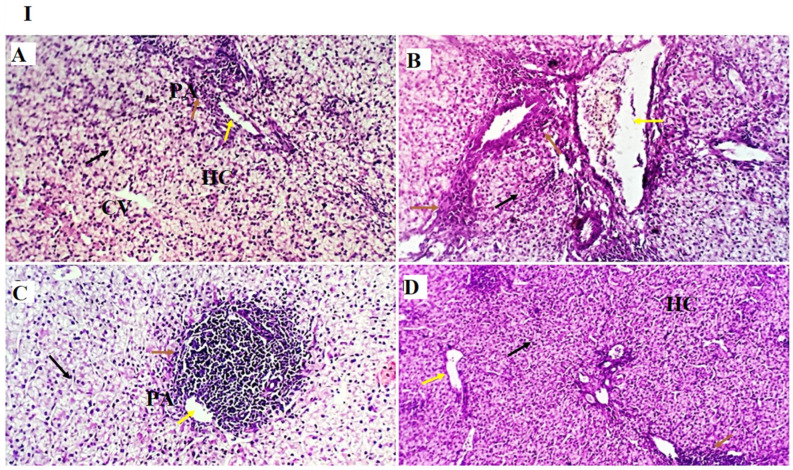

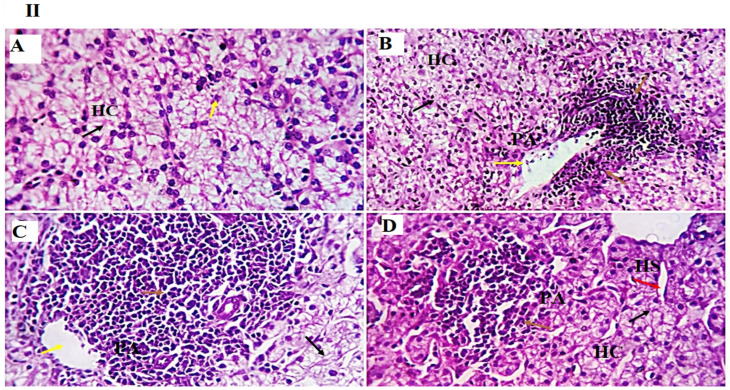
(**I**,**II**)**.** Photomicrographs from the liver ((**I**): H&E × 100 magnification, (**II**): H&E × 400 magnification) of chicken fed diet supplemented with 0 mg/kg (**A**), 50 mg/kg (**B**), 100 mg/kg (**C**), and 150 mg/kg (**D**) of GLC showing standard histological structure, including the portal area (PA, yellow arrow), hepatocytes (HC, black arrow) noted as a small mass around the central veins (CV), and a few to moderate round cells observed as a natural immune response around the portal area (PA, arrow). The 150 mg/kg group showed mild dilation of hepatic sinusoids (HS, red arrow).

The examined liver sections from the GLC0, GLC50, GLC100, and GLC150 groups showed the standard histomorphological structure of the liver. Mild portal vascular dilatation was observed in the GLC100 group. Moderate peri-portal lymphocytic aggregation were observed in GLC50, 100, and 150 (Figure 1I,II)).

### 3.7. Immunohistochemical Analysis

**Figure 2:**
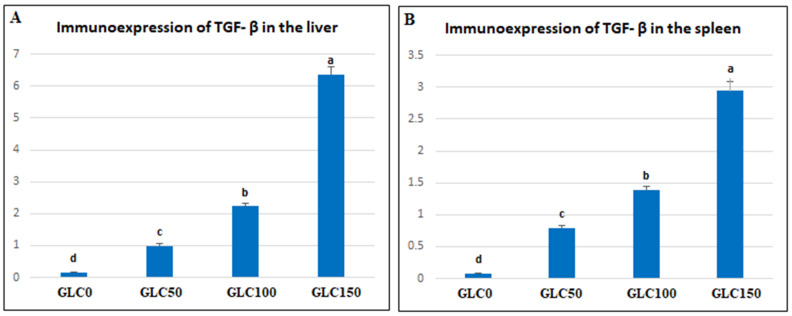
TGF-β morphometric analysis data in the liver (**A**) and spleen (**B**) of the experimental groups: 0 mg/kg (GLC0), 50 mg/kg (GLC50), 100 mg/kg (GLC100), and 150 mg/kg (GLC150). Mean values with different superscripts (^a, b, c, d^) in the same row differ significantly at *p* < 0.05. TGF-β: transforming growth factor β.

**Figure 3:**
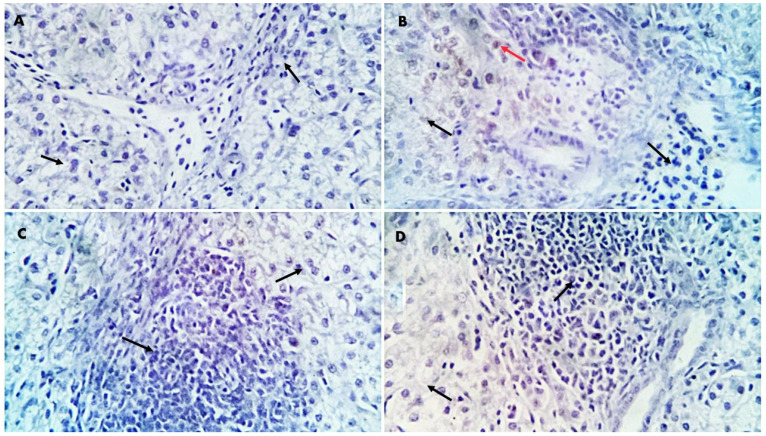
Positive and negative immunostained *TGF-β* cells (red and black arrows, respectively) in the liver sections. (**A**): GLC0, (**B**): GLC50, (**C**): GLC100, (**D**): GLC150. × 400 magnifications.

**Figure 4:**
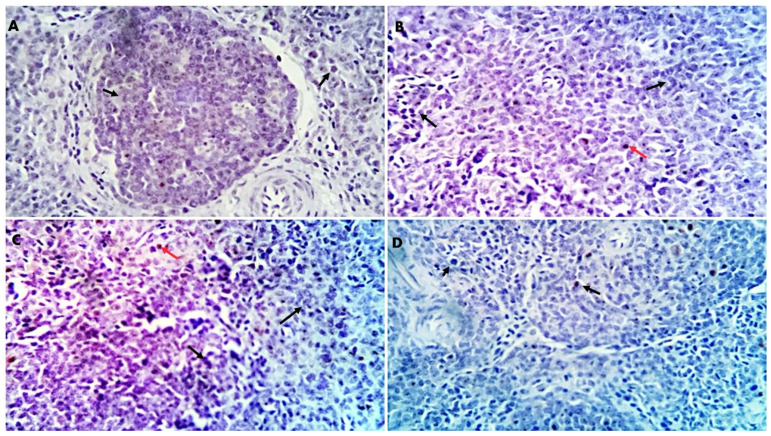
Positive and negative immunostained *TGF-β* cells (red and black arrows, respectively) in the spleen sections. (**A**): GLC0, (**B**): GLC50, (**C**): GLC100, (**D**): GLC150. × 400 magnifications.

Morphometric analysis of liver and spleen sections revealed significant upregulation in the percentages of positive cells per three high-power fields to the proinflammatory indicator (*TGF-β*) (*p* < 0.01) in the different GLC-supplemented groups as follows: 0.15%, 0.995%, 2.22%, and 6.36% for GLC0, GLC50, GLC100, and GLC150 groups, respectively, in the liver sections (Figure 2A and Figure 3), and 0.08%, 0.79%, 1.39%, and 2.94% for GLC0, GLC50, GLC100, and GLC150 groups, respectively, in the spleen sections (Figure 2B and Figure 4).

### 3.8. Economic Efficiency

Dietary GLC supplementation quadratically increased FC and TC at 50 and 100 mg kg^−1^ concentrations (*p* < 0.01). However, TR, NR, FC/kg gain, PI%, and EE were not affected by GLC supplementation (*p* > 0.05) (Table 7).

## 4. Discussion

GLC have important biological activities and play an important function in disease prevention and animal production performance [40]. In the present study, birds fed GLC-enriched diets grew at the same rate as the GLC0 group. In contrast, the GLC50 and GLC100 groups showed an increase in their total feed intake. This is supported by the fact that the energy contribution of GLC is used for immunity and other physiological functions, such as antioxidant activity, rather than bird growth. Similarly, Chae et al. [41] reported that GLC-fed birds consumed more feed than nonsupplemented birds. Zhang et al. [42] noted no significant effect of 1,000 mg kg^−1^ GLC (derived from *Agrobacterium* sp.) on birds’ growth. Cox et al. [8] showed no significant differences in the growth of broilers fed diets supplemented with 200 or 1000 mg glucans kg^−1^ diet with or without an *Eimeria* challenge. Zhang et al. [16] demonstrated an increased average daily gain and improved FCR in broiler chickens fed β1,3/1,6-glucan-supplemented diets. Zhang et al. [43] reported enhanced BWG and FCR of heat-stressed chicks by increasing energy digestibility. Chae et al. [41] found increased BWG of chickens fed finisher diets containing 200 and 400 mg kg^−1^ GLC (derived from *Saccharomyces cerevisiae*). In contrast, Kazempour et al. [44] reported that GLC supplementation (30 g kg^−1^) reduced feed intake and daily average gain and improved FCR in broiler chickens during the starter, grower, and finisher periods.

In the current study, dietary GLC supplementation did not influence the carcass traits; dressing percentage; and the weights of the spleen, liver, intestine, and gizzard. These results indicate that GLC supplementation had no negative effects on the internal organs of birds. The bursal weight was decreased at the highest GLC concentration of 150 mg kg^−1^; this may be due to increased inflammatory response. Similarly, Tang et al. [45] reported no effect of GLC on the carcass features of Peking ducks. In the study of Kazempour et al. [44], GLC at 30 g kg^−1^ concentration had no effect on the internal organs, namely the liver, spleen, and pancreas of broiler chickens; however, it decreased the abdominal fat and increased the weight of the small intestine and gizzards.

Many factors affect meat quality, such as diet composition, genetic structure, sex, housing management, slaughter method, and the type of muscle fibers [46]. The recent trend in poultry consumption demonstrates the significance of meat quality assurance in poultry establishments [47]. The chemistry of chicken meat is important because it affects storage and processing. Scarce information is available on the effect of 1,3-β-glucans on the fatty acid content of broiler muscles. Hence, it was difficult to compare the present results with previous reports. In the present study, dietary GLC changed the fatty acid profile of breast muscles. In the breast muscles of chickens fed GLC-supplemented diets, the percentages of α-linolenic acid, eicosapentaenoic acid, docosapentaenoic acid, docosahexaenoic acid, linoleic acid, arachidonic acid, total n-3, and total n-6 PUFA continued to increase in a concentration-dependent manner. Our results suggested that dietary GLC may affect lipid metabolism. The n-3 and n-6 PUFA play critical roles in human nutrition. They are precursors to various biological compounds, including prostaglandins, eicosanoids, and thromboxanes, which regulate the activity of the immune and cardiovascular systems [48]. Salah et al. [49] found increased n-3 PUFA content in the breast muscles of birds fed a synbiotic-supplemented diet. In the current study, however, GLC addition had no effect on breast muscle composition as measured by the content of dry matter, crude protein, fat, and ash. These findings support the growth performance results and demonstrate that the energy contribution of GLC was not used for the bird’s growth or muscle formation. Tang et al. [45] demonstrated no significant changes in the chemical composition (crude protein, fat, and ash) and fatty acid content in the meat of Peking ducks fed Sophy β-glucans-supplemented diet in comparison with the control and the bacitracin zinc groups.

Regarding the liver and kidney function tests, the current study showed that the GLC150 group had higher serum ALT and uric acid levels, while their levels did not stray from the normal levels [50]. Moreover, no differences in serum AST and creatinine levels were noted between the groups. These results may be attributed to the mild inflammatory responses caused by the highest level of GLC (150 mg kg^−1^), as observed in the results of the liver histopathological examination, where normal liver histoarchitecture was found in all experimental groups with mild portal vascular dilatation and lymphoplasmacytic aggregations in the GLC100 group. A minimal number of inflammatory cells were recorded in the GLC0 and GLC50 groups. The GLC150 group showed moderate peri-portal lymphocytic aggregation.

Regarding the antioxidant status of broiler chickens in response to dietary GLC supplementation, the results showed increased serum levels of TAC, CAT, and SOD. The MDA levels in GLC-fed groups were decreased in a concentration-dependent manner. GLC can boost the antioxidant capacity of animals by enhancing the activity of the antioxidant enzymes CAT, SOD, and glutathione peroxidase, as well as by enhancing the scavenging activity of the superoxide anion and the hydroxyl radical and by preventing lipid peroxidation [40]. The combination of the hydroxyl groups of GLC and the metal ion inhibits the generation of hydroxyl radicals and prevents lipid peroxidation [51,52]. Guo et al. [53] reported that GLC protects the cells of the porcine jejunum epithelial cell line IPEC-J2 cells from oxidative stress induced by deoxysqualenol by reducing the levels of reactive oxygen species and MDA and by upregulating the glutathione level. Our results proposed that GLC can provide a protective effect against oxidative stress by stimulating the antioxidant system. Liu et al. [54] discovered that the addition of yeast cell wall components to the broiler chicken diet increased the levels of intestinal reduced glutathione and glutathione reductase.

Regarding the inflammatory responses in broiler chickens fed with a GLC-supplemented diet, the results showed increased serum levels of IL-1β and IFN-γ in the GLC-fed groups in a concentration-dependent manner. Moreover, dietary GLC supplementation increased TGF-β immunoexpression in chicken liver and spleen tissues in a concentration-dependent manner. Proinflammatory cytokines (interferon-γ and IL-1β), which are essential immune proteins, are endogenous signaling molecules that facilitate cellular defense against inflammatory response [55]. Broiler chickens fed diets supplemented with GLC showed improved cell-mediated [41,56] and humoral immune responses [15,16]. The ability of GLC to enhance the immune response establishes their possible use as an antibiotic alternative by preventing numerous economically critical pathogens, for instance, *Escherichia coli* and *Salmonella enterica* [19,57]. GLC may activate macrophages or monocytes by inducing the major histocompatibility complex (MHC) compound [16]. Immune potentiation occurs because of this activity, which includes the activation of T-helper and natural killer cells, cytotoxic macrophages, T-cell development and differentiation, and the initiation of the alternative complement pathway [58]. The other signaling messengers, such as IL-1, IL-2, IFN-γ, and TNF-α, may also be included in the immuno-regulating system. The results of liver histopathology confirmed these findings, wherein moderate-to-high peri-portal lymphocytic aggregation was observed in the GLC100 and GLC150 groups. The accumulation of portal inflammatory cells appears to be a highly resistant and defensive mechanism rather than a destructive inflammatory process.

From an economic point of view, dietary GLC supplementation did not affect the TR, NP, performance index, and EE of the treated groups. This effect was due to the increase in the amount of feed intake by birds in the GLC50 and GLC100 groups with no change in the growth rate of birds. However, birds fed GLC-enriched diets have the advantages of transferring energy to the development of immune response and antioxidant capability, which improves the health and performance of birds. These results have more economic value than weight gain in maintaining adequate health of chicken flocks because clinical and subclinical diseases result in reduced feed intake, weight, and severe morbidity and mortality, which consequently harm animal welfare and cause substantial economic problems to poultry producers [13,59].

## 5. Conclusions

Based on the study outcomes, we can conclude that dietary GLC can be used as a feed additive in the diet schedule of broiler chickens at a concentration of up to 100 mg kg^−1^ with no adverse effects on growth, carcass traits, and internal organs. The addition of GLC improves the antioxidant status of the fed birds by enhancing the activity of antioxidant enzymes, namely CAT, SOD, and glutathione peroxidase. GLC also boosts immunity by enhancing the release of proinflammatory cytokines. Furthermore, GLC supplementation helps enrich breast muscles with n-3 and n-6 PUFA. However, a limitation of this study is that it used mixed chickens and only five replicates per treatment. Therefore, using mono-sex chickens and a higher number of replications in future studies is recommended.

## Figures and Tables

**Table 1:** The proximate chemical composition of the basal diet as fed basis (%).

Ingredients	Starter	Grower	Finisher
Corn 7.5% Cp	55.8	59.3	62.3
Soybean meal 47% CP	33.7	28.1	23.7
Corn gluten meal 60% CP	3.75	5.30	5.90
Oil (soya)–e76	2.20	3.00	4.00
Dicalcium phosphate dcp 18%	1.50	1.40	1.30
Calcium carbonate	1.20	1.20	1.10
Sodium bicarbonate	0.250	0.250	0.250
Dl methionine 99%	0.400	0.300	0.330
Broiler premix *	0.300	0.300	0.300
L-Lysine HCL 98%	0.470	0.450	0.400
Salt	0.150	0.150	0.150
Antimycotoxin	0.100	0.100	0.100
Choline 60 veg	0.070	0.070	0.070
L-Threonine 98.5%	0.100	0.100	0.100
Enzyme Phytase	0.005	0.005	0.005
Chemical analysis (%)			
Dry matter	88.7	88.8	88.9
ME poultry (kcal/kg)	3000	3100	3200
Crude protein	23.1	21.5	20.0
Methionine	0.710	0.600	0.610
Lysine	1.46	1.30	1.14
Calcium	0.930	0.910	0.820
Av. Phosphorus	0.470	0.430	0.400

* Premix per kg of diet: Vit. A, 1500 IU; Vit. D3, 200 IU; Vit. E, 10 mg; Vit. K3, 0.5 mg; folic acid, 0.55 mg; thiamine, 1.8 mg; pantothenic acid, 10 mg; riboflavin, 3.6 mg; niacin, 35 mg; pyridoxine, 3.5 mg; biotin, 0.15 mg; cobalamin, 0.01 mg; Fe, 80 mg; Mn, 60 mg; Zn, 40 mg; Cu, 8 mg; I, 0.35 mg; Se, 0.15 mg. CP: crude protein.

**Table 2:** Effects of dietary GLC on the growth of birds.

Parameters	GLC0	GLC50	GLC100	GLC150	SEM	*p* Value	
						Linear	Quadratic
Initial BW (g)	99.16	98.41	98.71	98.58	0.15	0.37	0.39
Starter period							
BW (g)	323	334	332	329	1.82	0.29	0.09
BWG (g)	223	236	233	231	1.84	0.25	0.07
FI (g)	257	256	259	262	2.22	0.50	0.83
FCR	1.15	1.08	1.11	1.13	0.01	0.86	0.15
Grower period							
BW (g)	1141	1171	1194	1143	13.77	0.84	0.25
BWG (g)	818	836	862	814	12.68	0.93	0.30
FI (g)	1073 ^b^	1182 ^ab^	1215 ^a^	1111 ^ab^	18.79	0.29	<0.01
FCR	1.31	1.42	1.41	1.36	0.02	0.44	0.12
Finisher period							
BW (g)	1911	2021	2033	2006	23.41	0.22	0.21
BWG (g)	769	851	838	863	14.53	0.06	0.34
FI (g)	1331 ^b^	1597 ^a^	1561 ^ab^	1476 ^ab^	34.79	0.14	0.01
FCR	1.73	1.88	1.87	1.71	0.04	0.87	0.13
Overall performance							
BW (g)	1911	2021	2033	2008	23.41	0.22	0.21
BWG (g)	1811.54	1923.20	1934.30	1908	23.43	0.22	0.21
FI (g)	2660 ^b^	3036 ^a^	3035 ^a^	2849 ^ab^	52.20	0.16	<0.01
FCR	1.46	1.58	1.57	1.49	0.02	0.75	0.07
PER	3.25	3.04	3.05	3.21	0.04	0.76	0.06
RGR	180.2	181.4	181.4	181.2	11.70	0.15	0.16

Mean values with different superscripts (^a^, ^b^) in the same row differ significantly at *p* < 0.05. BW: body weight, BWG: body weight gain, FI: feed intake, FCR: feed conversion ratio, PER: protein efficiency ratio, RGR: relative growth rate.

**Table 3:** Effects of dietary GLC supplementation on the characteristics of carcass, gut, and immune organs (%).

Parameters	GLC0	GLC50	GLC100	GLC150	SEM	*p* Value	
						Linear	Quadratic
Dressing	59.53	59.11	59.95	59.87	0.55	0.75	0.89
Intestine	5.22	4.85	5.82	5.51	0.21	0.36	0.95
Spleen	0.12	0.08	0.09	0.12	0.01	0.97	0.07
Bursa	0.15 ^a^	0.12 ^ab^	0.14 ^a^	0.09 ^b^	0.01	0.01	0.39
Gizzard	2.24	2.27	2.09	2.08	0.11	0.58	0.94
Liver	2.82	2.01	2.41	2.34	0.13	0.38	0.17

Mean values with different superscripts (^a^, ^b^) in the same row differ significantly at *p* < 0.05.

**Table 4:** Effects of dietary GLC on the composition and fatty acid profile of breast muscles.

Parameters	GLC0	GLC50	GLC100	GLC150	SEM	*p* Value	
						Linear	Quadratic
Dry matter% ^1^	26.57	28.63	28.11	28.14	0.63	0.23	0.20
Crude protein% ^2^	65.93	66.6	66.43	66.36	2.40	0.81	0.74
Fat% ^2^	5.53	5.3	6.33	5.46	0.40	0.82	0.70
Ash% ^2^	6	5.33	4.33	5.66	0.58	0.54	0.19
α-linolenic acid (18:3 n-3)	0.03 ^b^	0.04 ^ab^	0.053 ^ab^	0.056 ^a^	0.006	<0.01	0.56
Eicosapentaenoic acid (20:5 n-3)	0.02 ^c^	0.026 ^bc^	0.036 ^ab^	0.04 ^a^	0.003	<0.01	0.9
Docosapentaenoic acid (22:5 n-3)	0.02	0.03	0.036	0.04	0.006	<0.01	0.66
Docosahexaenoic acid (22:6 n-3)	0.01 ^b^	0.02 ^ab^	0.026 ^a^	0.03 ^a^	0.003	<0.01	0.5
Linoleic acid (18:2 n-6)	0.79 ^b^	0.81 ^ab^	0.83 ^ab^	0.84^a^	0.02	<0.01	0.6
Arachidonic acid (20:4 n-6)	1.18 ^b^	1.23 ^ab^	1.27 ^a^	1.26 ^a^	0.01	<0.01	0.11
n-3 (% of total fatty acids)	0.09 ^b^	0.12 ^ab^	0.15 ^ab^	0.16 ^a^	0.03	<0.01	0.48
n-6 (% of total fatty acids)	1.98 ^b^	2.04 ^ab^	2.10 ^a^	2.11 ^a^	0.02	<0.01	0.19
n-3: n-6 ratio	0.05	0.07	0.06	0.07	0.004	0.41	0.24

Mean values with different superscripts (^a^, ^b^, ^c^) in the same row differ significantly at *p* < 0.05; ^1^ on fresh basis; ^2^ on dry matter basis. n-3 PUFA: omega-3 polyunsaturated fatty acids, n-6 PUFA: omega-6 polyunsaturated fatty acids.

**Table 5:** Effects of dietary GLC supplementation on serum biochemical parameters.

Parameters	GLC0	GLC50	GLC100	GLC150	SEM	*p* Value	
						Linear	Quadratic
ALT (U/L)	5 ^b^	6.67 ^ab^	6.33 ^ab^	7.67 ^a^	0.37	0.08	0.03
AST (U/L)	43.33	51.66	54.66	55.33	2.31	0.07	0.39
Creatinine (mg/dL)	0.23	0.25	0.24	0.25	0.004	0.07	0.34
Uric acid (mg/dL)	3	3.1	3.56	3.76	0.13	0.03	0.48

Mean values with different superscripts (^a^, ^b^) in the same row differ significantly at *p* < 0.05. ALT: alanine aminotransferase, AST: aspartate aminotransferase.

**Table 6:** Effects of dietary GLC on the serum antioxidant and inflammatory indices.

Parameters	GLC0	GLC50	GLC100	GLC150	SEM	*p* Value	
						Linear	Quadratic
MDA (nmol/mL)	7.03 ^a^	4.53 ^b^	3.55 ^b^	3.68 ^b^	0.47	<0.01	0.03
SOD (U/mL)	137.32 ^c^	145.60 ^bc^	153.97 ^ab^	160.29 ^a^	2.84	<0.01	0.72
CAT (U/mL)	3.96 ^c^	4.73 ^bc^	5.67 ^ab^	6.45 ^a^	0.32	<0.01	0.99
TAC (U/mL)	10.25 ^c^	11.51 ^b^	13.03 ^a^	13.64 ^a^	0.41	<0.01	0.19
IFN- γ (pg/mL)	6.73 ^c^	10.83 ^b^	12.93 ^ab^	14.43 ^a^	0.91	<0.01	0.07
IL1β (μg/mL)	148 ^b^	161 ^a^	162.67 ^a^	169 ^a^	2.52	<0.01	0.20

Mean values with different superscripts (^a^, ^b^, ^c^) in the same row differ significantly at *p* < 0.05. MDA: malondialdehyde, SOD: superoxide dismutase, CAT: catalase, TAC: total antioxidant capacity, IFN-γ: interferon-gamma, IL-1β: interleukin-1β.

**Table 7:** Effect of dietary GLC supplementation on economic efficiency.

Parameters	GLC0	GLC50	GLC100	GLC150	SEM	*p* Value	
						Linear	Quadratic
TR (USD/bird)	2.44	2.58	2.59	2.56	0.033	0.22	0.21
TC (USD/bird)	1.74 ^b^	1.94 ^a^	1.94 ^a^	1.85 ^ab^	0.029	0.10	<0.01
FC (USD/bird)	1.33 ^b^	1.52 ^a^	1.53 ^a^	1.44 ^ab^	0.029	0.10	<0.01
NP (USD/bird)	0.69	0.64	0.65	0.70	0.026	0.87	0.40
FC/kg gain (USD/bird)	0.73	0.79	0.79	0.75	0.012	0.55	0.07
PI%	130.10	128.47	129.76	134.58	2.68	0.60	0.61
EE	1.91	2.47	2.39	2.06	0.128	0.74	0.12

Mean values with different superscripts (^a^, ^b^) in the same row differ significantly at *p* < 0.05. FC: feed cost, TC: total cost, TR: total return, NP: net profit, PI: performance index, EE: economic efficiency.

## Data Availability

The datasets generated or analyzed during the current study are not publicly available but are available from the corresponding author upon reasonable request.
